# Boosting the Electrochemical Performance of Polyaniline by One-Step Electrochemical Deposition on Nickel Foam for High-Performance Asymmetric Supercapacitor

**DOI:** 10.3390/polym14020270

**Published:** 2022-01-10

**Authors:** Syed Shaheen Shah, Himadri Tanaya Das, Hasi Rani Barai, Md. Abdul Aziz

**Affiliations:** 1 Interdisciplinary Research Center for Hydrogen and Energy Storage (IRC-HES), King Fahd University of Petroleum & Minerals, KFUPM Box 5040, Dhahran 31261, Saudi Arabia; syedshaheenshah3@gmail.com; 2 Physics Department, King Fahd University of Petroleum & Minerals, KFUPM Box 5047, Dhahran 31261, Saudi Arabia; 3 Centre of Excellence for Advance Materials and Applications, Utkal University, Bhubaneswar 751004, India; Himadritanaya1991@gmail.com; 4 School of Mechanical and IT Engineering, Yeungnam University, Gyeongsan 38541, Korea

**Keywords:** electrochemical deposition, polyaniline, nickel foam, jute derived activated carbon, asymmetric supercapacitor, high-performance

## Abstract

Energy generation can be clean and sustainable if it is dependent on renewable resources and it can be prominently utilized if stored efficiently. Recently, biomass-derived carbon and polymers have been focused on developing less hazardous eco-friendly electrodes for energy storage devices. We have focused on boosting the supercapacitor’s energy storage ability by engineering efficient electrodes in this context. The well-known conductive polymer, polyaniline (PANI), deposited on nickel foam (NF) is used as a positive electrode, while the activated carbon derived from jute sticks (JAC) deposited on NF is used as a negative electrode. The asymmetric supercapacitor (ASC) is fabricated for the electrochemical studies and found that the device has exhibited an energy density of 24 µWh/cm^2^ at a power density of 3571 µW/cm^2^. Furthermore, the ASC PANI/NF//KOH//JAC/NF has exhibited good stability with ~86% capacitance retention even after 1000 cycles. Thus, the enhanced electrochemical performances of ASC are congregated by depositing PANI on NF that boosts the electrode’s conductivity. Such deposition patterns are assured by faster ions diffusion, higher surface area, and ample electroactive sites for better electrolyte interaction. Besides advancing technology, such work also encourages sustainability.

## 1. Introduction

The exponential growth of industries and advancement in electronics has evolved society to a point of being technology dependent. With the present-day demand for efficient energy sources, there is a need for clean and sustainable energy. Researchers and industrialists are taking revolutionary actions to prevent environmental pollution. In making advanced energy storage devices for future electronics, lightweight, inexpensive, less hazardous chemical, and biodegradable electrodes have been highly explored [1,2,3]. However, constructing such nanomaterials is still challenging on small laboratories and a large industrial scale. Due to such issues, researchers have developed electrodes from natural precursors or ecofriendly materials [4,5]. Besides that, developing electrode materials for batteries and supercapacitors is energy and time-consuming, which ultimately hike the cost of devices in the energy market. Therefore, developing electrode materials with a facile, cost-effective, time-efficient, and eco-friendly approach is quite pertinent.

Recently, electrodes for supercapacitors have been investigated extensively for their benefits such as high power density, fast charging/discharging, and longer durability [6]. The design pattern of supercapacitors can be classified into symmetric, asymmetric, and hybrid supercapacitors [3,7]. The type of electrode materials utilized as positive and negative electrodes in supercapacitors determines the type of device designed. When similar electrodes are used, it is called a symmetric supercapacitor, where two different electrodes are used to form an asymmetric supercapacitor (ASC). Similarly, when a battery-type electrode is used, it is known as a hybrid supercapacitor [3,8].

Electrode materials can behave like electric double-layer capacitors (EDLCs), pseudocapacitors, or battery-type depending upon the charge storage mechanism in the electrodes. Generally, carbon materials—such as activated carbon, graphene, and carbon nanotubes—store charge by the surface phenomenon, i.e., by adsorption/desorption of electrolyte ions at the electrode–electrolyte interface, and so they are considered EDLC materials [9]. On the other hand, metal oxides, conductive polymers, and chalcogenides undergo redox reactions, and so they are considered pseudocapacitors or battery-type electrode materials [10]. An electrode can be a promising candidate if it exhibits efficient electrochemical properties such as high electrical conductivity, high thermal/chemical stability, high specific charge storage ability, and high energy and power densities [6].

Owing to the unique features of conductive polymers, they have gained attention in energy storage devices [11,12]. Polymers are usually synthesized by polymerization of their monomers by different methods [13]. The electrochemical polymerization of monomers has been an attractive way to generate polymers easily in less time [14]. The parameters governing the amount and morphology of electrodeposited polymers are surface substrate, electrochemical bath, bath concentration, deposition technique, and deposition time [9,15]. Similar parameters also affect the growth and structure for the polymerization of aniline to polyaniline (PANI) [16]. Zhang et al. deposited PANI on Ag/CN in a solution of 0.1 M aniline and 0.1 M H_2_SO_4_ with a deposition current density of 5 mA/cm^2^ at different timings from 5 to 20 min with 5 min interval [17]. Interestingly, Yu et al. deposited PANI on 3D-graphene by using 1 M HClO_4_ and 0.1 M aniline as electrolyte and the as-prepared 3D-graphene as a working electrode by galvanostatic electrodeposition at 0.1 A/g [18]. Electrodeposition of PANI has led to the development of lightweight and compact electrodes for advanced supercapacitors like flexible and micro-supercapacitors [19,20]. Substrate selection plays an essential role in the growth of nanomaterials—such as stainless steel, nickel foam (NF), and carbon cloth—which are generally used as a substrate for the deposition of electrode materials. Among these, NF is the best performing substrate, as it gives a large surface area and network structure that provides nanomaterials to participate in electrochemical performance actively. For instance, Shaikh et al. deposited PANI on stainless steel and achieved a superior specific capacitance of 757 F/g [21]. Where Zhong et al. deposited PANI on activated carbon cloth which delivered a specific capacitance of 369 F/g [22]. Xie et al. deposited PANI/Graphene on Ni foam and showed a high specific capacitance of 261 F/g with high energy and power densities of 23.2 Wh/kg and 399 W/kg [23]. Whereas Ruan et al. synthesized PANI layers/carbon layer coating on Ni foam by electrodeposition which delivered a high specific capacitance of 610 mF/cm^2^ with high energy and power densities of 41 μWh/cm^2^ and 0.9 mW/cm^2^ [24]. Moreover, this work is found to be a promising candidate for developing high-performing ASCs in less time with the utilization of less hazardous chemicals and in a cost-effective manner.

The application of biomass-derived activated carbon as electrodes in symmetric and ASCs are highly investigated. Various forms of biomass are generated from different sources of plants or animals [25,26]—such as human hair derived carbon flakes which showed high specific capacitance of 340 F/g [27]; and rice husk-derived carbon, which delivered a capacitance of 197.6 F/g [28]. Even though the resources for the activated carbon are sufficient, there are still challenges for optimizing the synthesis of particular nanomaterials, the morphology of carbon or carbon composite obtained, along with the purity of the samples. Therefore, researchers are exploring various techniques to synthesize activated carbon in an eco-friendly and cost-effective approach. Especially when bio-wastes are converted to resources, the accumulation of waste is reduced and it provides a more economical approach to generating biodegradable clean energy. The carbon was derived from flour food waste and utilized as a supercapacitor electrode which showed a high specific capacitance of 278 F/g [29]. Similarly, carbon derived from mung bean husks exhibited a high specific capacitance of 390 F/g [30].

In this context, the present work has chosen conductive polymer PANI electrodeposited on NF. The binder-free electrode was implemented as a positive electrode in the ASC device. Where jute sticks derived activated porous carbon nanosheets (JAC) were obtained and used as the negative electrode. The asymmetric device was fabricated with the prepared electrodes, PANI/NF and JAC/NF, and 3 M KOH aqueous electrolyte and the corresponding electrochemical properties were investigated.

## 2. Experimental

### 2.1. Materials

All the chemicals were used in pure forms as purchased. Aniline monomer, sodium bicarbonate (NaHCO_3_), sulfuric acid (H_2_SO_4_), potassium hydroxide pellets (KOH), polyvinylidene fluoride (PVDF), and N-methyl-2-pyrrolidone (NMP) were purchased from Sigma Aldrich Inc., ST. Louis, MO, USA. NF (0.5 mm, 110PPI) which was obtained from the Minihua store on AliExpress.com. Jute sticks were gathered from the western part of Mominpur, Keshabpur, Jessore, Bangladesh. Nitrogen (N_2_) gas with high purity was supplied by Specialty Gases Company Limited, Jubail, Kingdom of Saudi Arabia. 

### 2.2. Synthesis of Jute Sticks Derived Activated Carbon Nanosheets (JAC)

The negative electrode was synthesized from biomass (jute sticks) to obtain eco-friendly and bio-degradable nanomaterials. The JAC were synthesized from the jute sticks in a similar approach to our earlier work [25]. Briefly, the biomass jute sticks were cut into small pieces and cleaned with de-ionized (DI) water followed by drying at 100 °C for 24 h in an electric oven. The dried pieces were crushed to fine powders and mixed with the activating agent (NaHCO_3_) in a ratio of 1:4. Then the mixture was subjected to carbonization at 850 °C for 5 h under the N_2_ atmosphere in a tubular furnace with a heating and cooling rate of 10 °C/min and 5 °C/min, respectively. To remove other impurities from pyrolyzed biomass that may be formed due to activation, the obtained carbon powder was acid-treated with 0.5 M HCl two times, washed with DI water two times, and then filtered. Then filtered carbon powder was dried at 60 °C for 12 h in an electric oven, and JAC was obtained.

### 2.3. Electrochemical Deposition of PANI on Nickel Foam (PANI/NF)

**Figure 1:**
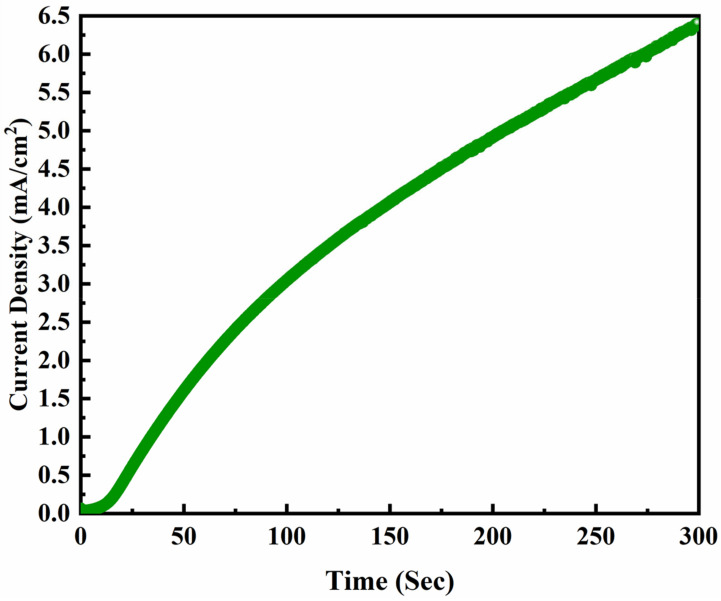
Amperometric curve of PANI electrodeposited on NF substrate at +0.85 V vs. Ag/AgCl for 300 s.

An electrochemical deposition process was considered to synthesize positive electrode materials. The conductive polymer PANI was deposited by the amperometric method with the current–time transient. The operational parameters were optimized at deposition potential +0.85 V vs. Ag/AgCl and deposition time was 300 s. The deposition area of PANI on NF was optimized to 1 cm^2^ for effective growth to be implemented as a positive electrode in the supercapacitor. The electrochemical solution for the deposition was taken the same as our earlier experiment, i.e., 0.5 M aniline and 1 M H_2_SO_4_ in a three-electrode set-up [14]. The substrate NF was used as a working electrode, Ag/AgCl as a reference electrode, and Pt wire as a counter electrode. The electrochemical polymerization aniline monomer was observed to have greenish blue color deposition on bare NF substrate. This can be substantiated by the increasing current amperometric curve given in Figure 1. Uniform growth and strongly adhered binder-free PANI on NF was obtained as PANI/NF.

### 2.4. Characterization of the As-Synthesized Electrodeposited PANI/NF

The electrodeposition of PANI on NF as substrate was analyzed by advanced characterization techniques. The morphological study of PANI/NF was performed using high-resolution field-emission-scanning-electron-microscopy (FESEM) TESCAN-LYRA-3, Tescan, Brno, Czech Republic. Energy dispersive X-Ray analysis (EDAX) was carried out with an Oxford instruments Xmass detector equipped with the FESEM. The crystallization and purity of deposited PANI/NF was examined by X-ray diffraction (XRD) analysis utilizing a Rigaku Miniflex-II diffractometer, Cu-K-Alpha radiations (λ = 0.15416 nm) operating at a constant current of 30 mA and a constant voltage of 40 kV. Transmission electron microscopy (TEM: JEOL JEM 2100F, Tokyo, Japan) was employed to visualize the surface morphology of the PANI nanoparticles. The X-ray photoelectron spectroscopy (XPS) equipped with an Al-K-alpha monochromatic X-ray source (ESCALAB-250Xi XPS-Microprobe, Thermo-Scientific, Waltham, MA, USA) was employed for the elemental composition of the prepared PANI/NF.

### 2.5. Fabrication of Working Electrodes and ASC Cell Assembly 

The prepared PANI/NF electrode was directly used as a positive electrode. The negative electrode was fabricated by mixing 90 wt % of the JAC and 10 wt % of PVDF as a binder. JAC was dissolved in NMP via. stirring at 80 °C, followed by the slow addition of PVDF into the solution. The stirring was continued for 5 h until a homogeneous slurry was attained, which was then cast on a NF (working area of 1 cm^2^; used as a current collector). Subsequently, the JAC/NF electrode was dried in an electric oven for 12 h at 70 °C. The PANI/NF//JAC/NF ASC was assembled with two electrodes in the sandwich-type cell assembly, where JAC/NF was used as a negative electrode, and PANI/NF was used as a positive electrode. The PANI/NF//JAC/NF ASC was assembled with two working electrodes separated by a filter paper separator soaked in a 3 M KOH aqueous electrolyte.

### 2.6. Electrochemical Measurements

The electrochemical measurements like cyclic voltammetry (CV), galvanostatic charge–discharge (GCD), and electrochemical impedance spectroscopy (EIS) of the fabricated ASC were performed with a CHI-760E potentiostat. The ASC performances with PANI/NF as positive and JAC/NF as negative electrode were tested in 3 M KOH electrolyte. The fabricated device’s areal capacitance (*C*, F/cm^2^) was measured from the CV curves by applying Equation (1) [14,25,31,32,33]

(1) $$C=\frac{\int I\times dV}{A\times \nu \times \Delta V}$$

where *∫I × dV* represents the area under CV curve (in watt), A stands for the working area of two electrodes (in cm^2^), *ν* is the scan rate (in volt/sec), and ∆*V* is the operating potential window (OPW in volt). Similarly, the areal capacitance was also calculated from GCD measurements using Equation (2) [14,25,31,32,33]

(2) $$C=\frac{I\times \Delta t}{A\times \Delta V}$$

where *I* is the constant current (in ampere), ∆*t* represents the time (sec) for discharge, *A* represents the working area of two electrodes (in cm^2^), and ∆*V* represents the discharge OPW (V).

The energy (*E*, Wh/cm^2^) and power density (*P*, W/cm^2^) are very important performance parameters of the supercapacitor device and can be estimated in accordance with Equations (3) and (4), respectively [14,25,31,32,33].

(3) $$E=\frac{1}{2}\frac{C\times \Delta V^{2}}{3.6}$$

(4) $$P=\frac{E\times 3600}{\Delta t}$$

## 3. Results and Discussion

### 3.1. Morphological and Structural Analysis of the Bare NF and Electrodeposited PANI/NF

#### 3.1.1. FESEM

**Figure 2:**
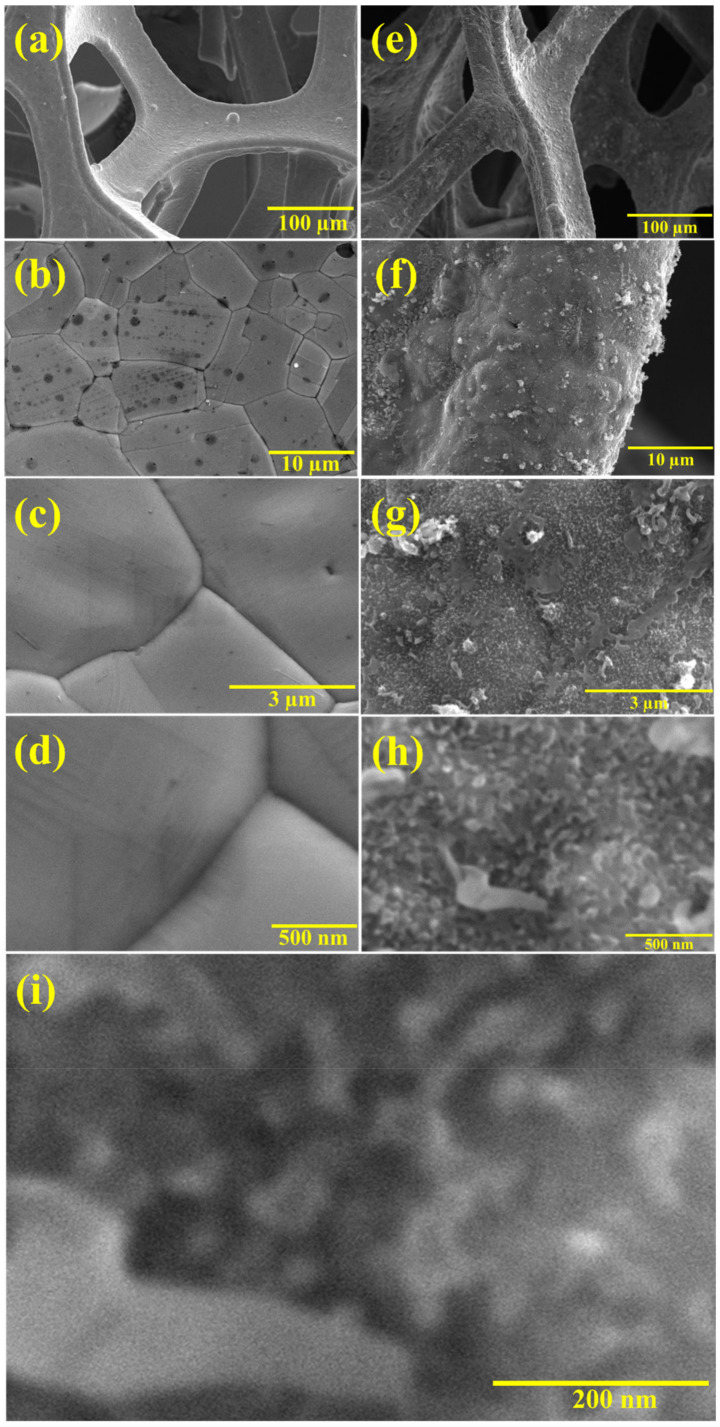
FESEM micrographs of (**a**–**d**) bare NF and (**e**–**i**) PANI/NF at different magnifications.

Morphological investigation is one of the best parameters to understand the prepared samples and their growth. Figure 2 shows FESEM images of the NF and PANI/NF synthesized by the electrodeposition method. Figure 2a–d shows the FESEM images of the bare NF at different magnifications, while Figure 2e–i shows the FESEM images of the PANI/NF at different magnifications. The NF as the substrate has provided a well-connected porous platform for uniform and wide distribution of PANI on it. This design helped in saving time in fabricating electrodes. The PANI on the NF-network seemed to be well distributed and adhered to the NF that could exhibit good conductivity and stability. Additionally, the electrodes can be designed binder-free as PANI is directly deposited on the NF. The neatly deposited PANI with NF can be an efficient electrode for supercapacitor applications. Similar PANI electrodeposition is seen in other reports for PANI electro-polymerization [18].

#### 3.1.2. EDAX and Elemental Mapping

**Figure 3:**
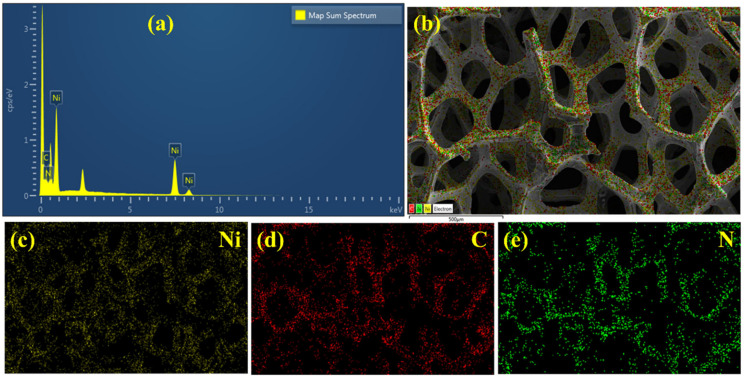
(**a**) EDAX spectrum, (**b**) FESEM micrograph used for EDAX analysis of PANI/NF and the corresponding elemental mappings of (**c**) Ni, (**d**) C, and (**e**) N.

The elemental analysis and mappings of the PANI/NF were obtained by EDAX, as shown in Figure 3. Figure 3a shows the EDAX spectrum of PANI/NF, where the respective peaks corresponding to the elements carbon, nitrogen, and nickel are detected in the corresponding area of PANI/NF shown in Figure 3b. Ni peaks, appearing in the EDAX spectrum of PANI/NF, are due to the NF used as a substrate. The unmarked peak at around 2.2 keV is for sulfur due to the incorporation of sulfate ions during the electrochemical deposition of PANI in the H_2_SO_4_ acid electrolyte bath. Whereas no extra elements were observed, indicating no impurities in the prepared PANI/NF. The elemental mapping images, shown in Figure 3c–e, reveal the uniform spatial distribution of the elements (Ni, C, and N) present in the PANI/NF.

#### 3.1.3. XRD 

**Figure 4:**
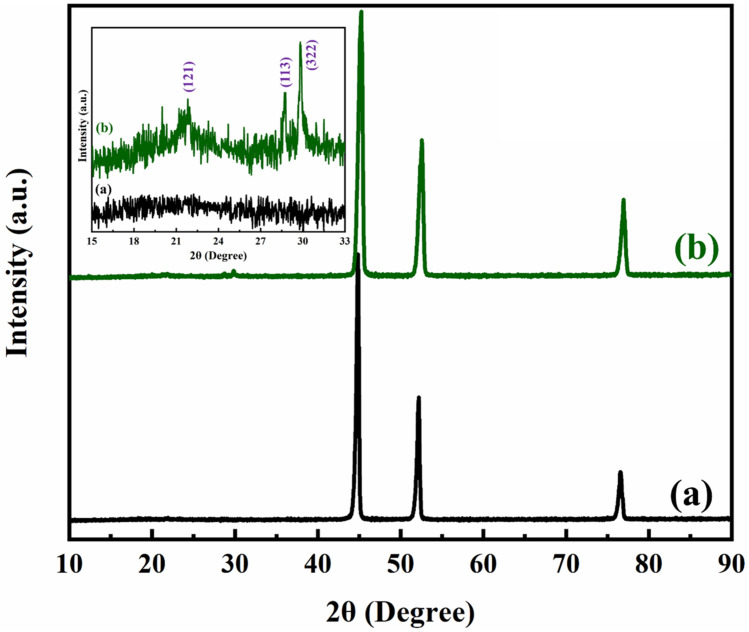
XRD spectrum of (**a**) bare NF and (**b**) PANI/NF. The inset shows an enlarged region overview from 2θ = 15° to 33°.

The electro-polymerization of aniline in a strong acidic bath leads to unreacted spices/ions that can accumulate on the surface of PANI/NF. XRD analysis was done to determine the purity, phase, and crystallinity of PANI on NF. Figure 4 shows the XRD patterns for NF and PANI/NF. The major 2θ diffraction peaks obtained for pure NF at around 47°, 52°, and 78° are ascribed to (111), (200), and (220), respectively [34]. The smaller peaks found around 15° to 30° (as shown in the inset of Figure 4) confirmed the deposition of PANI on the NF. PANI, being semi-crystalline, showed three sharp peaks at 2θ around 22°, 28°, and 30° corresponding to 121, 113, and 322 diffraction planes, respectively. The XRD peaks of PANI exhibited good agreement with those in the literature [35,36] and agreed with the JCPDS card no. 72–0634. Thus, the XRD spectrum revealed the purity and fine polymerization of aniline to PANI by amperometric method at deposition potential +0.85 V vs. Ag/AgCl for 300 s.

#### 3.1.4. TEM

**Figure 5:**
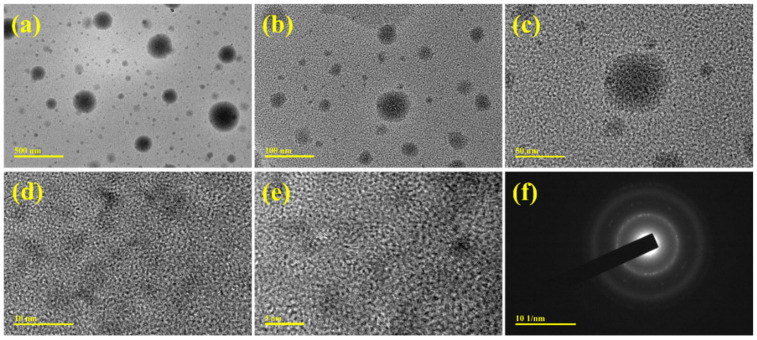
(**a**–**c**) TEM images, (**d**,**e**) HTREM images, and (**f**) the corresponding SAED pattern of PANI nanoparticles.

TEM analysis was performed to investigate the morphology of the prepared PANI further. To obtain dispersed nanoparticles of PANI for TEM analysis, the PANI/NF was ultrasonicated in ethanol for ~5 h. Figure 5a–c shows TEM images of the PANI at different magnifications, revealing nanoparticles with average particle sizes of ~45 nm (ImageJ software was used to find the average particle size). The TEM and FESEM images show a clear difference between the PANI morphology, this could be due to the longer ultrasonication time of the electrochemically prepared PANI/NF during PANI dispersion in ethanol to prepare the TEM sample. The lattice fringes observed in the high-resolution TEM images (HRTEM) (Figure 5d,e) demonstrate the high degree of crystallinity of the PANI nanoparticles, as also evident in the XRD result (Figure 4b). The nanoparticles in the TEM/HRTEM images are seen to possess a spherical morphology. In Figure 5e, lattice fringes emanating from the PANI nanoparticle were recorded within a single nanoparticle, which suggests the formation of irregular quasi-crystaline nanoparticles. Furthermore, the selected area electron diffraction (SAED) pattern of PANI nanoparticles (Figure 5f) indicates the presence of distinct diffractions, with the appearance of dark and bright fringes showing the crystalline structure of the PANI.

#### 3.1.5. XPS

**Figure 6:**
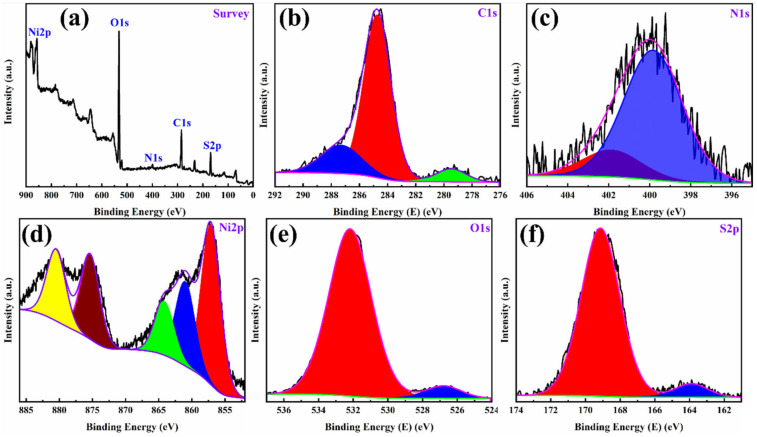
(**a**) XPS survey spectrum of PANI/NF and high resolution deconvoluted XPS spectrum of (**b**) C1s, (**c**) N1s, (**d**) Ni2p, (**e**) O1s, and (**f**) S2p.

XPS is one of the advanced techniques to indicate the oxidation states present in the sample and to understand the functionalities for faradaic and non-faradaic performance during charge storage [37]. The XPS survey scan spectrum for PANI/NF electrode discovered only five elements—i.e., S, C, N, O, and Ni—on the electrode’s surface, as shown in Figure 6a. Figure 6b–f shows the deconvoluted spectra for the C1s, N1s, Ni2p, O1s, and S2p, respectively. The C1s peak is found with major three binding energies at 280.8, 284.7, and 288.5 eV. These binding energies established the existence of C–C, C–N, and C–O/C = O bonds in PANI on the NF substrate surface. The high-resolution N1s spectrum of the PANI/NF found with binding energies of 398.9, 399.9, and 401.6 eV indicate the presence of = N^-^, –NH^–^, and N^+^ groups, respectively. The Ni2p indicates metallic nickel for the NF substrate. The various peaks show a minimum amount of nickel oxides and hydroxides on the surface due to exposure of NF to moisture. The high-resolution O1s spectrum of the PANI/NF electrode shows peaks at 531.6 eV and 527.8 eV, indicating the C = O bond and C–OH/C–O–C bonds, respectively. The presence of the S2p state in the electrode specified the incorporation of SO_4_^2^^–^ ions during the electrochemical deposition of PANI in the H_2_SO_4_ acid electrolyte bath. Thus, the XPS substantiated with the XRD and FESEM studies, confirming the purity phase of PANI obtained by electrochemical polymerization on NF.

### 3.2. Electrochemical Analysis of the Electrodeposited PANI/NF and PANI/NF//JAC/NF ASC 

**Figure 7:**
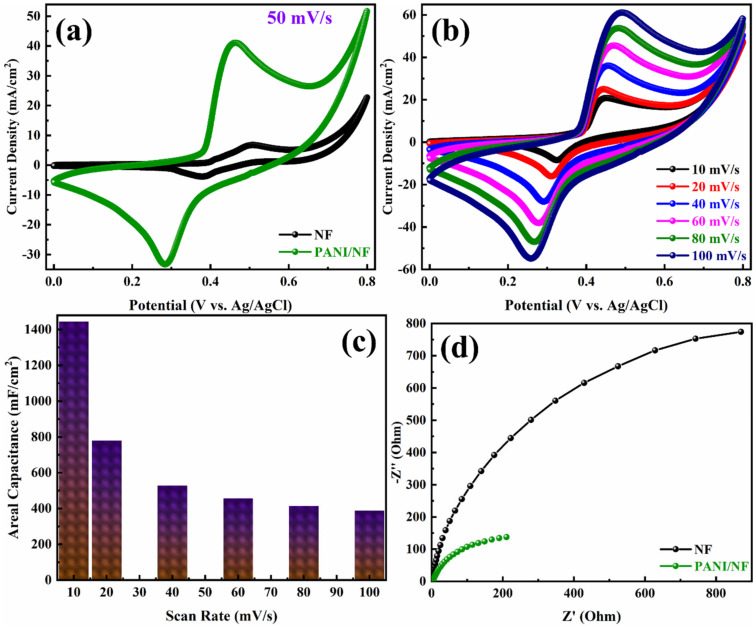
Cyclic voltammograms of (**a**) bare NF and PANI/NF at a scan rate of 50 mV/s, (**b**) PANI/NF at different scan rates. (**c**) Areal capacitance of the PANI/NF electrode as a function of the scan rate and (**d**) Nyquist plots of bare NF and PANI/NF in the three-electrode system.

Electrochemical tests were performed in a three-electrode electrochemical cell with a 3 M KOH aqueous solution as the electrolyte, platinum wire as a counter electrode, and a Ag/AgCl as a reference electrode to evaluate the electrochemical performance of the PANI/NF electrode. Figure 7a compares cyclic voltammograms acquired at a scan rate of 50 mV/s on bare NF and PANI/NF electrodes. The PANI/NF electrode had a significantly higher current density than the NF electrode, showing that the PANI/NF electrode had a superior capacitive performance. Figure 7b shows the CV curves of the PANI/NF electrode acquired at various scan rates ranging from 10 to 100 mV/s. Each curve shows a pair of distinct redox peaks (anodic and cathodic peaks) that correspond to the reversible Faradic redox processes, demonstrating the usual pseudocapacitive behavior of the PANI [38,39]. PANI has received interest as an organic conducting polymer due to its unique physical and chemical features such as ease of synthesis, electrochromism, environmental friendliness, outstanding redox properties, ease of doping/de-doping, high conductivity, and high surface area [40]. Furthermore, the excellent symmetry of the anodic and cathodic peaks indicates the PANI/NF electrode’s redox reversibility. The PANI/NF electrode demonstrated good areal capacitance, a large integrated area, and exceptional rate capacity, with scan rates ranging from 10 to 100 mV/s. It appeared to favor fast ionic transport rates, with a remarkable capacitance at high scan rates. It was difficult to determine the exact mass of the electrochemically deposited PANI on NF; thus, we calculated the areal capacitances instead of specific capacitances. The PANI/NF electrode exhibits large areal capacitances, as illustrated in Figure 7c, with the highest areal capacitance of 1445 mF/cm^2^ at a scan rate of 10 mV/s. Furthermore, the PANI/NF electrode has good rate capability, with high capacitance retention when the scan rate increases from 10 to 100 mV/s. The enhanced deposition of electrochemically active PANI on NF is responsible for the PANI/NF electrode’s remarkable electrochemical performance. EIS of the bare NF and PANI/NF electrodes was performed in a three-electrode electrochemical cell using 3 M KOH as an electrolyte to further illustrate the feasibility of the PANI/NF electrode as a highly conductive and active electrode for supercapacitors. Figure 7d shows the Nyquist plots of bare NF and PANI/NF electrodes depict a minor solution resistance at the high-frequency region. Charge transfer resistance (R_ct_) is an essential parameter to determine the electrochemical performance of an electrode material for supercapacitors [41]. Due to the highly conductive nature of PANI, the PANI/NF bares very low R_ct_ as compared to the bare NF. The low resistance may be generated due to adhering deposition of PANI on NF. The binder-free deposition improves the conductivity and provides a better electrode/electrolyte interaction. Such electrode design might have increased the wettability of the PANI/NF electrode in the 3 M KOH electrolyte.

**Figure 8:**
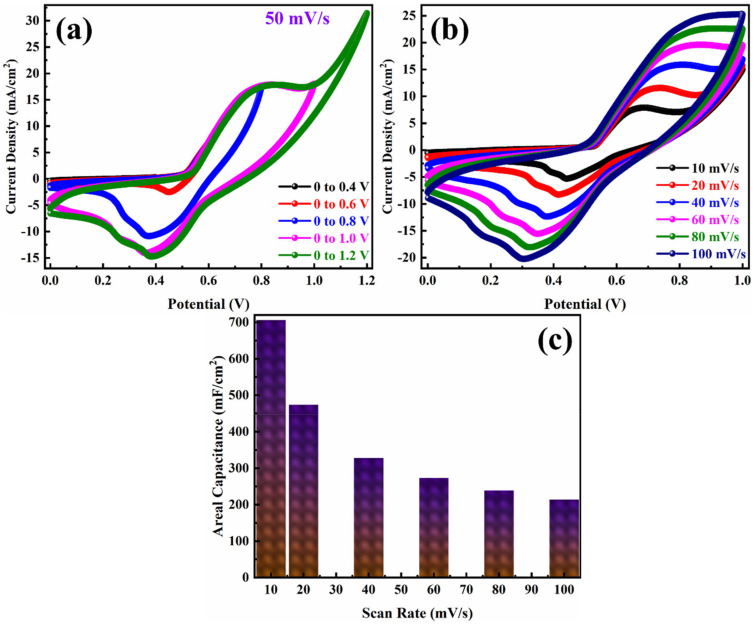
Cyclic voltammograms in (**a**) different potential windows at a scan rate of 50 mV/s and (**b**) in the optimum potential window from 0 to 1 V at different scan rates, and (**c**) the corresponding areal capacitances obtained from the cyclic voltammograms of PANI/NF//JAC/NF ASC.

To evaluate the electrochemical properties of ASC were measured by fabricating the device with as-prepared PANI/NF as the positive electrode and JAC/NF as the negative electrode in 3 M KOH. The PANI/NF stores charge in the redox method while the JAC behaves as an EDLC electrode. The combination of electrodes in the ASC device was optimized by various techniques like CV, GCD, and EIS. The cyclic voltammogram measurements for PANI/NF//JAC/NF ASC were carried out in 3 M KOH electrolyte, as shown in Figure 8. The CV curves obtained for different potential window helps to set the device best operating potential. Figure 8a shows the CV curves of the PANI/NF//JAC/NF ASC in different OPWs recorded at a scan rate of 50 mV/s. The trend of CV curves is similar for all potential range 0 to 1.0 V, where a sharp increase in the current density between 1.0 V and 1.2 V shows an unusual behavior. Therefore, CV curves at different scan rates in the OPW from 0 to 1.0 V were acquired. Figure 8b shows the CV curves, which represent an increase in current density with an increase in scan rates. The areal capacitance of the ASC was calculated from the CV curves at different scan rates by using Equation (1). The calculated areal capacitances at different scan rates 10, 20, 30, 40, 60, 80, and 100 mV/s are 706, 474, 328, 273, 238, and 214 mF/cm^2^, respectively and represented in Figure 8c. Such high charge storage by the ASC device may be possible due to well device fabrication and compatible PANI and JAC electrodes in KOH electrolyte. Even in a high scan rate of 100 mV/s the ASC device stored a high amount of charge, stating good rate capability for the device. Better retention ability and electrochemical reversibility can also be observed in constant CV curves. Small humps acquired in the CV curves indicate the redox phenomenon of the charge storage mechanism during the charge/discharge process. It is seen that the areal capacitance reduces on an increase of scan rates. On the increase of scan rates, the charge transfer is superficial and faster, so the electrode/electrolyte interaction reduces, resulting in lower areal capacitance [42]. Such behavior is usually seen in supercapacitors, as reported earlier [43]. 

**Figure 9:**
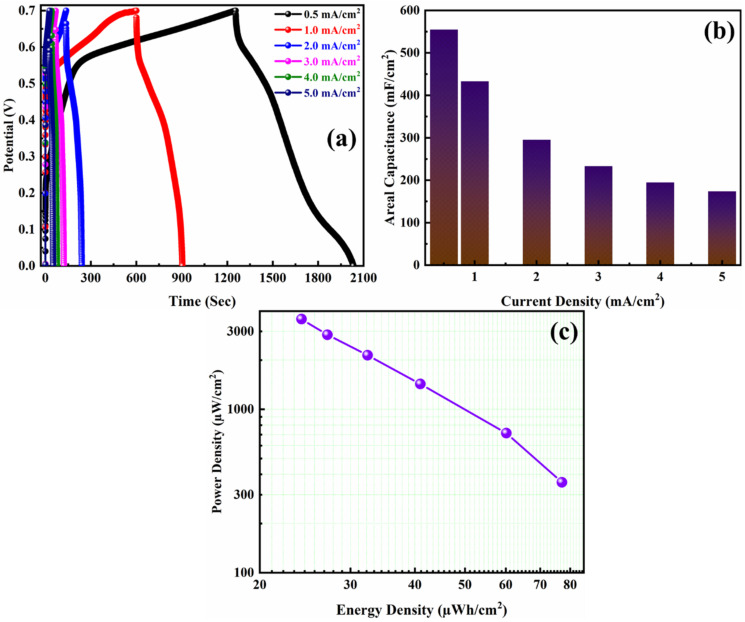
(**a**) GCD profiles at different current densities, (**b**) the corresponding areal capacitances obtained from the GCD profiles, and (**c**) Ragone plot of the PANI/NF//JAC/NF ASC.

Furthermore, the GCD studies were performed on the ASC in 3 M KOH at different current densities in a potential window 0 to 0.7 V. The GCD profile resembles the charge/discharge behavior as seen in the CV curves. The plateau-like GCD curves with small humps stand for the redox reaction during the electrochemical process. Figure 9a shows the GCD profiles for different current densities for PANI/NF//JAC/NF ASC. The ASC device exhibited high areal capacitance as 555 F/ cm^2^ at 0.5 mA/ cm^2^ current density. At 1, 2, 3, 4, and 5 mA/cm^2^ current densities, the areal capacitance calculated from GCD profiles are 433, 295, 233, 195, and 174 mF/cm^2^. Figure 9b shows a gradual decrease in areal capacitance with increased current densities substantiating the performances as an ideal supercapacitor. A negligible IR-drop is found, suggesting a well-performing ASC with low resistance and high charge storage. The conductive polymer PANI might have increased conductivity, and the NF provided a platform for large electrode/electrolyte interaction. The PANI was uniformly deposited on the NF substrate, where NF provided a high porosity and large surface area for electrodeposition of PANI. The PANI/NF electrode’s network structure would have allowed faster charge transfer kinetics that ultimately reduced the IR-drop and boosted the charge storage in the device. On the other hand, the efficiently activated carbon electrode (JAC/NF) can compensate for the conductive polymer and result in better electrochemical performance.

The device can be only feasible if it can deliver a high energy and power density. Generally, ASC device exhibits high energy and power density. The energy and power density for the fabricated PANI/NF//JAC/NF ASC was estimated from the GCD profile by the Equations (3) and (4). Figure 9c shows the Ragone plot stating the energy and power density outcome for the PANI/NF//JAC/NF ASC. The device delivered an energy density of 77 µWh/cm^2^ at a power density of 357 µW/cm^2^. The device was able to outcome a high-power density of 3571 µW/cm^2^ at 5 mA/cm^2^. The calculated values for all the energy and power densities at different current densities have been tabulated in Table 1. 

**Figure 10:**
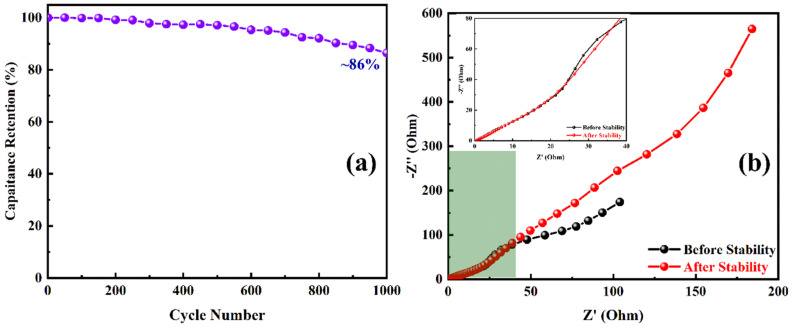
(**a**) Cyclic stability up to 1000 GCD cycles and (**b**) Nyquist plots before and after the stability of the PANI/NF//JAC/NF ASC. The inset of (**b**) shows the enlarged portion of the Nyquist plots at the high-frequency region.

Additionally, the ASC device stability is essential for practical applications in the electronic industry. The PANI/NF//JAC/NF ASC was subjected to 1000 charge/discharge cycles for stability test. It was found that the device displayed good stability. The capacity retention for ASC was obtained around ~86% even after 1000 cycles, as shown in Figure 10a. The EIS is a vital electrochemical analysis technique where the electrode performance in the electrolyte can be well understood. The study reveals the solution resistance, charge transfer resistance, and type of reaction that occur during the electrochemical process [44]. Figure 10b shows the Nyquist plot depicts a minor solution resistance at the high-frequency region; low semicircle at mid-frequency region gives minimal intrinsic resistance of the electrode material; and electrode/electrolyte interface where nearly straight line represents the Warburg component at low-frequency region. The inset in Figure 10b shows the Nyquist plots at a higher frequency. The low resistance may be generated due to adhering deposition of PANI on substrate NF. The binder-free deposition improves the conductivity as well as provides a better electrode/electrolyte contact. Such electrode design might have increased the wettability of the PANI/NF electrode in the 3 M KOH electrolyte. The impedance study for before and after cycle life represents the stability of the ASC device. Thus, the results confirmed that the asymmetric behavior of the PANI/NF//JAC/NF boosts supercapacitive performance with high stability and high energy and power densities. The fabricated ASC (PANI/NF//JAC/NF) was relatively worthy as per earlier reported supercapacitor work given in a comparative Table 2.

## 4. Conclusions

A boost in electrochemical performance in ASCs was obtained due to the resourceful electrodes. The deposition of conductive polymer PANI network on the NF played a role model for the device designing. In summary, we have successfully electrodeposited PANI on NF and assembled an ASC with KOH electrolyte. High porosity and active surface area provided by NF has led to more electrode–electrolyte interaction for the electrodeposited PANI/NF as a binder-free electrode. The developed PANI/NF//JAC/NF ASC achieved a maximum areal capacitance of 555 mF/cm^2^ at a current density of 0.5 mA/cm^2^ in 3 M KOH with energy and power density values of 77 µWh/cm^2^ and 357 µW/cm^2^, respectively. This charge storage ability of PANI/NF as a positive electrode was supported by the conductive carbon nanosheets derived from biomass (jute sticks). Both the electrodes have facilitated the fast charge transfer and low internal resistance, thus contributing to high-capacity retention and stability of the ASC. A high energy and power density was obtained for the fabricated device. Thus, such cost-effective, easy means can also be implemented to design electrodes for the batteries or solar cells. Bio-mass derived electrodes also contribute to developing clean and sustainable energy.

## Figures and Tables

**Table 1:** Areal capacitances, energy densities, and power densities of the PANI/NF//JAC/NF ASC.

From CV Curves		From GCD Profiles			
Scan Rate (mV/s)	Areal Capacitance (mF/cm^2^)	Current Density (mA/cm^2^)	Areal Capacitance (mF/cm^2^)	Energy Density (µWh/cm^2^)	Power Density (µW/cm^2^)
10	706	0.5	555	77	357
20	474	1.0	433	60	714
40	328	2.0	295	41	1429
60	273	3.0	233	32	2143
80	238	4.0	195	27	2857
100	214	5.0	174	24	3571

**Table 2:** Comparison of the fabricated PANI/NF//JAC/NF ASC with the reported PANI-based ASCs in the literature.

Positive Electrode	Negative Electrode	Electrolyte	Capacitance (F/g or F/cm^2^)	Current Density or Scan Rate	Energy Density	Power Density	Capacitance Retention (%) @ Cycles	Ref.
GQDs	PANI	H_3_PO_4_–PVA	210 μF/cm^2^	15.0 μA/cm^2^	0.029 μWh/cm^2^	7.46 μW/cm^2^	85.6 % @ 1500	[45]
PANI/graphene	PANI/graphene	H_3_PO_4_/PVA	261.24 F/g	0.38 A/g	23.2Wh/kg	399 W/kg	89% @ 1000	[23]
CNT/PANI	CNT/PANI	PVA–H_2_SO_4_	680 mF/cm^2^	1 mA/cm^2^	…	…	100% @ 500	[46]
PANI@CNT/AWC	PANI@CNT/AWC	H_3_PO_4_-PVA	1019.5 F/g	10 mA/cm^2^	40.5 Wh/kg	162.5 W/kg	93.74% @ 10000	[47]
PANI/cellulose/PANI	PANI/cellulose/PANI	1M H_2_SO_4_	1079 F/g	1.73 A/g	100.9Wh/kg	12.1 kW/kg	86% @ 2100	[48]
PANI-LiPF_6_	PANI-LiPF_6_	Et_4_NbF_4_	107 F/g	1.25 mA/cm^2^	…	…	78.5% @ 9000	[49]
PANI	PANI	H_3_PO_4_-PVA	282 F/g	2.5 A/g	…	…	55% @ 200	[50]
PANI/CNT/PEO	PANI/CNT/PEO	1M H_2_SO_4_	385 F/g	0.5 A/g	7.11 Wh/kg	201 W/kg	81.4% @ 1000	[51]
BPO–PANI	BPO-PANI	0.5 M H_2_SO_4_	361 F/g	0.25 A/g	…	…	72.2 @ 500	[52]
PANI/CoSe_2_/NF	AC/NF	6 M KOH	434 F/g	0.25 A/g	118 Wh/kg	462 W/kg	82% @ 10000	[53]
GH/PANI	GH/PANI	1 M H_2_SO_4_	710 F/g	2 A/g	24 Wh/kg	30 kW/kg	86% @ 1000	[54]
rGO/PANI	rGO/PANI	1 M H_2_SO_4_	853.7 F/g	1 A/g	14.8 Wh/kg	6.7 kW/kg	92.6% @ 8000	[55]
PANI/NCNT	PANI/NCNT	PVA/H_2_SO_4_	128 F/g	2.47 A/g	11.1 Wh/kg	0.98 kW/kg	92% @ 10000	[56]
SP–PANI	SP-PANI	PVA/H_2_SO_4_	149.3 F/g	0.5 mA/cm^2^	13.0 μWh/cm^2^	0.40 mW/cm^2^	81.2% @ 5000	[57]
PANI/Ag/CNF	PANI/Ag/CNF	H_3_PO_4_-PVA	176 mF/cm^2^	10 mV/s	10.6 Wh/kg	225 kW/kg	…	[17]
PANI/NF	JAC/NF	3 M KOH	555 mF/cm^2^	0.5 mA/cm^2^	777 µWh/cm^2^	357 µW/cm^2^	86 @ 1000	This Work
